# Longitudinal study of the associations between change in sedentary behavior and change in adiposity during childhood and adolescence: Gateshead Millennium Study

**DOI:** 10.1038/ijo.2017.69

**Published:** 2017-05-09

**Authors:** K D Mann, L D Howe, L Basterfield, K N Parkinson, M S Pearce, J K Reilly, A J Adamson, J J Reilly, X Janssen

**Affiliations:** 1 Newcastle University, Institute of Health and Society, Newcastle upon Tyne, UK; 2 University of Bristol, School of Social and Community Medicine, MRC integrative Epidemiology Unit at Bristol University, Bristol, UK; 3 Newcastle University, Human Nutrition Research Centre, Newcastle upon Tyne, UK; 4 University of Strathclyde, School of Psychological Science and Health, Glasgow, Scotland

## Abstract

**Background:**

Sedentary time (ST) has been reported to have a range of negative health effects in adults, however, the evidence for such effects among children and adolescents is sparse. The primary aim of the study was to examine associations between changes in sedentary behavior (time and fragmentation) and changes in adiposity across childhood and adolescence.

**Methods:**

Participants were recruited as part of the Gateshead Millennium Study. Measures were taken at age 7 (*n*=502), 9 (*n*=506), 12 (*n*=420) and 15 years (*n*=306). Participants wore an ActiGraph GT1M and accelerometer epochs were ‘sedentary’ when recorded counts were ⩽25 counts per 15 s. ST was calculated and fragmentation (SF) was assessed by calculating the number of sedentary bouts per sedentary hour. Associations of changes in ST and SF with changes in adiposity (body mass index (BMI), and fat mass index (FMI)) were examined using bivariate linear spline models.

**Results:**

Increasing ST by 1% per year was associated with an increase in BMI of 0.08 kg m^−2^ per year (95% CI: 0.06–0.10; *P*<0.001) and FMI of 0.15  kg m^−2^ per year (0.11–0.19; *P*<0.001). Change in SF was associated with BMI and FMI (*P*<0.001). An increase of 1 bout per sedentary hour per year (that is, sedentary time becoming more fragmented) was associated with an increase in BMI of 0.07  kg m^−2^ per year (0.06–0.09; *P*<0.001) and an increase in FMI of 0.14  kg m^−2^ per year (0.10–0.18; *P*<0.001) over the 8 years period. However, an increase in SF between 9–12 years was associated with a 0.09  kg m^−2^ per year decrease in BMI (−0.18–0.00; *P*=0.046) and 0.11  kg m^−2^ per year decrease in FMI (−0.22–0.00; *P*=0.049).

**Conclusions:**

Increased ST and increased SF from 7–15 years were associated with increased adiposity. This is the first study to show age-specific associations between change in objectively measured sedentary behavior and adiposity after adjustment of moderate-to-vigorous-intensity physical activity in children and adolescents. The study suggests that, targeting sedentary behavior for obesity prevention may be most effective during periods in which we see large increases in ST.

## Introduction

Habitual sedentary time, and the fragmentation of sedentary behavior (the extent to which sedentary behaviors are prolonged or interrupted), have been reported to have important independent effects on all-cause mortality and cardiometabolic health in adults.^1, 2, 3, 4, 5, 6^ A recent systematic review of longitudinal studies suggested that time spent sedentary increases across childhood and adolescence, and sedentary time becomes more prolonged/less fragmented.^7^ In addition, sedentary behavior in childhood and adolescence may influence adult sedentary behavior by the establishment of long-term habits or norms. A recent systematic review suggested that early sedentary behavior tends to ‘track’ (tracking is the maintenance of relative position over time)^8^ and therefore it may be important to establish healthy lifestyles early on.

Recent systematic reviews and editorials have reported an increasing body of evidence on the health effects of sedentary behavior defined as screen time, but the evidence on the health impact of overall sedentary behavior during childhood and adolescence has only recently began to emerge and appears inconsistent.^9, 10, 11^ Studies have begun to identify possible effects of the number of breaks and bouts of sedentary behavior on cardiometabolic health in children and adolescents.^12, 13^ In a cross-sectional study of Canadian 8–11-year-olds, Saunders *et al.*^14^ found that greater fragmentation of sedentary time was associated with lower BMI *Z-*scores. Nevertheless, the evidence on associations between sedentary time or fragmentation and adiposity is limited to cross-sectional studies or studies of limited longitudinal duration. Recent reviews have found almost no evidence of associations between longitudinal changes in objectively measured sedentary behavior and adiposity across childhood and adolescence,^7, 15, 16, 17^ and so the hypothesis that objectively measured changes in sedentary behavior influences adiposity during childhood and adolescence remains largely untested, even though numerous research and policy interventions to modify sedentary behavior of children and adolescents are now being developed.

Given the limited evidence on the effect of objectively measured sedentary time and fragmentation, the main aim of the present study was to test for associations between changes in sedentary behavior (that is, sedentary time and fragmentation of sedentary time) and changes in adiposity across childhood and adolescence, in the Gateshead Millennium Study cohort. In addition, in a previously published study on this cohort the authors reported different rates of change in ST between the different time periods^18^ and therefore a secondary aim was to examine the effect of the rate of change in sedentary behavior on adiposity between measurement periods (that is, 7–9 years, 9–12 years and 12–15 years). The authors hypothesize that an increase in sedentary time results in an increase in adiposity and an increase in sedentary fragmentation results in a decrease in adiposity. In addition, it was hypothesized that the effect of the different rates of change during the different time periods may affect adiposity in different ways.

## Materials and methods

### Cohort

The Gateshead Millennium Study began as a prospective study of 1029 infants and their families recruited shortly after birth between June 1999 and May 2000 in Gateshead, an urban district in North East England. Full details of the study including recruitment and early life follow-up are described elsewhere.^19^ Physical activity and sedentary behavior measures in the cohort began in 2006–2007 when study participants were aged 6–8 years. For the present study four periods from when the children were age 6–15 years have been considered. Baseline measures for this study were taken between October 2006 and December 2007 (the first time physical activity and sedentary behavior measures were included), when the children were aged 6–8 years (‘7 years’); follow-up was conducted between October 2008 and September 2009, when the children were aged 8–10 years (‘9 years’); between October 2011 and September 2012, when the children were aged 11–13 years (‘12 years’), and between September 2014 and September 2015, when the adolescents were aged 14–16 years (‘15 years’). Written parental consent was obtained during each data collection period and the study was approved by the Gateshead and South Tyneside Local National Health Service Research Ethics Committee for data collection at 7 years and by the Newcastle University Faculty of Medical Sciences Ethics Committee for the 9-, 12- and 15-year data collections. Each child’s date of birth, sex and parental socio-economic position, measured by Townsend score (an area-based measure derived from the UK census in 1991), was recorded at birth. The measures described below were recorded at each of the 7-, 9-, 12- and 15-year baseline and follow-up periods.

### Body measurements

Height and weight were measured during baseline and follow-up periods. Height was measured to the nearest 0.1 cm using a Leicester portable height measure (Chasmors, London, UK). Weight (kg) and bio-impedance were measured while children wore light clothing using a Tanita TBF300MA. Body mass index (BMI) was calculated as weight (kg) divided by height (m) squared. Fat mass (kg) was derived using bio-impedance data to calculate age- and sex-specific total body water, hydration and lean mass as described by Lohman.^20^ The final calculation of fat mass comes from weight (kg) minus lean mass. Fat mass index (FMI) was calculated as fat mass (kg) divided by height (m) squared.

### Accelerometry—objective measurement of sedentary time and fragmentation of sedentary time

Sedentary behavior was measured using Actigraph GT1M accelerometers (ActiGraph Corporation; Pensacola, FL, USA) worn for 7 days during baseline and follow-up periods. Accelerometer protocols used in the Gateshead Millennium Study have been described in detail elsewhere.^21^ Briefly, participants recorded the times when the monitor was worn using a provided log sheet and non-wear time (including sleep) was removed manually based on the wear time diaries and visual inspection by a trained researcher. Non-wear time was not removed using consecutive zeros as it has been shown to affect the outcome significantly, especially in longitudinal studies in children and adolescents where changes in sedentary patterns are very likely.^22^ Data were collected in 15-s epochs and included in the analyses if participants had at least 3 days with ⩾6 h per day of accelerometer data; this has been shown to provide reliable estimates of sedentary behavior.^23^ Epochs were defined as sedentary when recorded counts were ⩽25 counts per 15 s. Sedentary time (ST) was expressed as the percentage of accelerometer wear time. A sedentary bout was defined as any period of time ⩾1 min of consecutive counts ⩽25 counts per 15 s.^24^ Fragmentation of sedentary time (SF) was quantified by calculating the number of sedentary bouts divided by total hours of sedentary time per day. This provides information about how ST is fragmented independent of overall ST; more bouts per sedentary hour indicate total ST is made up out of several shorter bouts. Along with ST and SF, moderate-to-vigorous-intensity physical activity (MVPA) was also measured using the accelerometers. Epochs were defined as MVPA if recorded counts were >800 counts per 15 s and expressed the percentage of accelerometer wear time.^21^

Participants were measured at the same time of the year during 7-, 9- and 12-year measures. However, some variation in measurement time occurred during the 15-year follow-up, therefore the month in which assessment took place was recorded. Season of measurement is defined in 4-month intervals of when measurements were taken; Winter: November, December, January and February; Autumn/Spring: March, April, September and October; Summer: May, June, July and August.

### Statistical analysis

Age, ST, SF, BMI, FMI, MVPA and Townsend score were treated as continuous variables whereas sex and season of measurement were treated as categorical. Multilevel linear spline random-effects models, with knot points at age 9 and 12 years, were used to describe the trajectory of change in ST, SF, BMI and FMI with age. ST, SF, BMI and FMI were repeatedly measured during baseline and follow-up periods, hence multilevel models with two levels (follow-up period (level 1) within each child (level 2)) were used. These models estimate individual-specific trajectories with no restriction on the number of measures, account for the correlation between repeated measures on the same child and allow for a change in scale and variation over time.^25, 26^ Four separate multilevel linear spline models were fitted with ST, SF, BMI and FMI as outcomes and knot points at 9 and 12 years each with a random intercept and a single-random slope (to capture individual deviation from the average trajectory, across all splines). Knot points were chosen at 9 and 12 years to allow for investigation of change between follow-up periods and following the recommendation that if there are only a small number of data collection time points the knot points may be placed at the mean follow-up age.^26^ In a previous study in this cohort differences in change of ST and SF were found between boys and girls.^18^ To account for these differences in the change of ST, SF, BMI and FMI all models included adjustment for sex over time. To attenuate regression coefficients for potential confounding of physical activity, all models also included adjustment for MVPA; interactions of sex and MVPA over time were also tested and included if found significant. Socio-economic status was included in all models as a potential confounder, however, it was found not to be associated with the change outcome nor did it attenuate regression coefficients and was therefore excluded in final models. The final linear spline random-effects models for the change in ST or SF over age included adjustment for sex and MVPA (including interactions of sex and MVPA over time) and seasonality. The final linear spline random-effects models for the change in BMI or FMI over age included adjustment for sex and MVPA.

To investigate the associations of changes in sedentary behavior with changes in adiposity we combined the final linear spline models for change in sedentary behavior (ST and SF) with the linear spline models for change in adiposity (BMI and FMI), as an extra response variable at the individual level, to form bivariate models. This was done for the linear spline random-effects model for change in ST as a predictor with change in BMI and change in FMI as the outcome (Model 1 and Model 2, respectively), as well as for the linear spline random-effects model for change in SF as a predictor with change in BMI and change in FMI as the outcome (Model 3 and Model 4, respectively). All bivariate models included covariate adjustments for sex, seasonality, MVPA and an interaction between sex and MVPA. Linear regression coefficients with 95% CI are reported for the association between sedentary behavior (ST and SF) at 7 years with adiposity (BMI and FMI) at 7 years, the association between sedentary behavior at 7 years with the change in adiposity and, finally, the change in sedentary behavior at 7 years with the change in adiposity.

Linear regression analysis was used to further investigate if the association between change in ST or SF and change in BMI or FMI differed between rates of change of follow-up age groups (age: 7–9 years, 9–12 years and 12–15 years) since these could not be estimated within bivariate models. Linear regression models considered all children with data at the specific time point with change in ST or SF as the outcome, and change in BMI or FMI as the independent predictor variable with adjustment for sex and change in MVPA over the same time period included.

## Results

During the four data collection periods 515, 517, 441 and 326 study members took part in the physical activity measurements. Of these 502 (97%), 506 (98%), 420 (95%) and 306 (94%) had valid adiposity data at 7, 9, 12 and 15 years, respectively (Table 1). There is a smaller sample with FMI measures due to missing (assumed to be at random) body composition data. Participants wore the accelerometer for an average of 671.1 min per day, 672.5 min per day, 7171.7 min per day and 725.6 min per day at age 7, 9, 12 and 15 years, respectively (Table 1).

Change trajectories for ST, SF, BMI and FMI are shown in Supplementary Tables 1–4. Briefly, ST increased from age 7 to 15 years by 188 min per day over the 8-year period, whereas fragmentation decreased by 3.7 bouts per sedentary hour (Table 1). Linear spline modeling showed a greater increase in ST between 9–12 years compared to 7–9 years and 12–15 years. The rate of change differed slightly between boys and girls (Supplementary Table 1). Fragmentation decreased most between ages 12 years and 15 years with again a slight difference between boys and girls (Supplementary Table 1). BMI and FMI increased from age 7 to 15 years (Table 1). BMI increased most between ages 9–12 years for boys and 7–9 years for girls and FMI increased most during the 9–12 years period in both boys and girls (Supplementary Table 1).

### Bivariate models

After adjustment for sex, MVPA and seasonality, higher levels of ST at baseline were associated with lower BMI and FMI (−0.05 kg m^−^^2^ for both) and with a decrease in BMI and FMI (−0.02 kg m^−^^2^ for both) between age 7 and 15 years (*P*<0.001 for all, Table 2). In addition, higher levels of SF at baseline were not associated with BMI (−0.002 kg m^−^^2^; *P*=0.968) and FMI (−0.01 kg m^−^^2^; *P*=0.927) or with a change in BMI (0.04 kg m^−^^2^; *P*=0.409) and FMI (0.05 kg m^−^^2^; *P*=0.396) between age 7 and 15 years (Table 2).

Associations between change in sedentary behavior and change in adiposity are shown in Table 2. After adjustment for sex, MVPA and seasonality a 1% increase in ST was associated with an increase in BMI of 0.08 kg m^−^^2^ per year (*P*<0.001). Increasing ST by 1% resulted in an increase in FMI of 0.15 kg m^−^^2^ (*P*<0.001). An increase of 1 bout per sedentary hour (that is, ST more fragmented) was associated with an increase in BMI of 0.07 kg m^−^^2^ and an increase in FMI of 0.14 kg m^−^^2^ (*P*<0.001 for both).

Associations between change in ST and change in FMI did not differ between time points. However, the association between ST and BMI differed slightly between time points. The results indicate a negative association (that is, increase in ST leads to decrease in BMI) from age 7 to 9 years (−0.02 kg m^−^^2^; *P*=0.053), whereas this is in the opposite direction for the periods 9–12 years (0.02 kg m^−^^2^; *P*=0.316) and 12–15 years (0.01 kg m^−^^2^; *P*=0.594). However, none of these associations were found significant (Tables 3 and 4). The association between change in SF and change in BMI and FMI differed between time periods (Tables 3 and 4). An increase in fragmentation (that is, 1 bout per sedentary hour more) was significantly associated with a 0.09 kg m^−^^2^ decrease in BMI and a 0.11 kg m^−^^2^ decrease in FMI over the 9–12 years period (*P*=0.046 and *P*=0.049, respectively). An increase in SF resulted in a 0.06 kg m^−^^2^ increase in BMI between 7 and 9 years (*P*<0.049) and no change in FMI (*P*=0.703). Finally, an increase in SF resulted a 0.05 kg m^−^^2^ increase in BMI (*P*=0.322) and a 0.04 kg m^−^^2^ increase in FMI (*P*=0.546) over the 12–15 years period. Associations between SF and BMI and FMI during the other time periods were non-significant (*P*>0.05).

## Discussion

### Main study findings and implications

In the present study, higher levels of ST at baseline were associated with lower BMI and smaller changes in BMI over time. However, increasing ST across childhood and adolescence was associated with an increase in body fatness from 7 to 15 years. Moreover, the association between increased ST and increased adiposity was independent of MVPA, suggesting that any impact of ST was not attributable simply to declining MVPA. The present study therefore provides the first longitudinal evidence that age-related increases in objectively measured sedentary time are associated with age-related increases in body fatness. In addition, the association between increased ST and increased adiposity was likely to be biologically as well as statistically significant: for example increasing ST by 3% per year (that is, equal to the average increase of ~24 min in this cohort) for 8 years will increase FMI by 0.147 × 3 × 8 =3.5 kg m^−^^2^. This equates to 1.3 kg of fat mass for a 15-year-old of average height in this cohort (that is, 167.0 cm) with mean fat mass of 18.4 kg. The present study is also is among the first to provide evidence of any adverse health impact of objectively measured increases in ST after adjustment for MVPA during childhood and adolescence.

Over the 8-year period, an increase in fragmentation of sedentary time was associated with an increase in FMI and BMI. This result contradicts the original study hypothesis and there are several reasons why this may be happening. First, the current study classified a bout of sedentary behavior as ⩾1 min of 100 c.p.m. which may have been too short. Saunders *et al.*^14^ showed a positive association between the number of bouts ⩾1–4 min and BMI *Z-*score in children with an obesity family history but negative associations for bouts ⩾5 min. Second, the results may have been affected by an unforeseen statistical bias or by possible confounders not included in the analysis (for example, diet). Further investigations indicated that when looking at the three different time periods (that is, 7–9 years, 9–12 years and 12–15 years) an increase in fragmentation was significantly associated with a decrease in FMI and BMI over the 9–12 years period. An increase in SF resulted in an increase in FMI and BMI between 7–9 years and 12–15 years however this was only significant with BMI as an outcome and change between 7–9 years of age (Tables 3 and 4). In addition, it has to be acknowledged that these regression analyses were cross-sectional and therefore the exact direction of the effect cannot be established.

There are several factors which may explain the associations between the changes in sedentary behavior and changes in adiposity observed in the present study. Sedentary behaviors, especially screen time, are often associated with opportunities to snack which may lead to increased energy intake while engaging in low energy expenditure activities resulting in a positive energy balance.^27^ In addition, engaging in sedentary behaviors such as television viewing or electronic gaming may adversely affect sleep which has been linked to an increase in adiposity.^28^

### Comparisons with other studies

Recent systematic reviews have noted the limited evidence on associations between objectively measured sedentary behavior and adiposity, and have called for more studies with stronger designs, including more longitudinal studies.^7, 15, 16, 17^ While previous studies have been small in number and inconclusive, the balance of available evidence has suggested that an association between sedentary behavior and adiposity would be unlikely. The current study showed the 9–12 years period to be a period in which the effects of increasing ST and lowering of SF appear to influence adiposity most. Few previous studies have included these periods. Most longitudinal studies have focused on specific periods such as childhood^20^ or adolescence^29, 30^ or included a wide range of ages (for example, 4–19 years).^31, 32^ In addition, different outcomes between studies may also be due to methodological decisions related to the accelerometer data. Several different cut points have been used to define sedentary behavior (that is, ranging from <100  c.p.m. to <1100  c.p.m.) and MVPA (that is, cut points ranging from >2295 c.p.m. to >3600 c.p.m.) as well as different non-wear criteria (ranging from 20 min of consecutive zeros to 60 min with and without allowance of interruptions).^21, 28, 29, 30, 31, 32, 33^ Previous research has shown that these differences affect the association with health outcomes and make comparison between studies difficult.^34^

Kwon *et al.*^33^ examined the longitudinal associations between ST and dual energy x-ray absorptiometry-measured body fat mass from age 8 to 15 years. The authors reported no associations between ST and fat mass after adjustments for MVPA. One of the reasons for the difference between the Kwon *et al.*^33^ study and the current study may be the method used to measure adiposity. The second study to examine the association between ST and change in BMI from age 9 to 15 years was the study by Mitchell *et al.*^35^ Mitchell *et al.*^35^ is the only study to date to suggest that accelerometer measured ST might be associated with adiposity, independent of MVPA. However, contradictory to the current study, the study by Mitchell *et al.* did not find a difference between time periods. In addition, Mitchell *et al.*^35^ reported that the associations between ST and BMI only applied to those with higher BMI and was revealed using quantile regression only. In the current study quantile regression was used to test whether associations between exposure and outcome values differed by the weight status of the participants. However, in this cohort no significant associations were found between ST and BMI or FMI in any of the weight status categories except the 75th percentile of BMI. In addition, the associations for SF and BMI or FMI were only significant for the 25th percentile (FMI) and the 50th percentile (BMI). However, while no significant results were found, the regression coefficients suggested the possibility of stronger associations between SF and adiposity in the higher weight status categories compared to the lower weight status categories (Supplementary Tables 2–5).

The evidence on the association between SF and adiposity is scarce in children and adolescents. Saunders *et al.*^14^ found that more breaks in ST were associated with lower BMI *Z-*score in a cross-sectional study of Canadian children aged 9 years. Interestingly, the authors also found a protective association between the number of sedentary bouts smaller than 5 min and BMI *Z-*score (that is, greater number of bouts was associated with lower BMI *Z-*scores). However, they did not report an association between the number of sedentary bouts ⩾5 min and BMI *Z-*score. Differences between studies may be the result of real biological differences between samples, and/or due to methodological differences.

### Study strengths and weaknesses

The main strengths of the present study were the novelty, the longitudinal design and analysis, the socio-economically representative and contemporary nature of the sample, the availability of high quality measures for both exposure and outcome variables, and the inclusion of relevant confounders. The longitudinal analysis made it possible to include all available data from all participants under a ‘missing at random’ assumption (that is, participants do not need valid data at all time points to be included in the analysis) which limits attrition. In a previous study within the same cohort we found that the availability of body fatness estimates was an important strength, as associations between exposures and outcomes which were observed with body fatness as the outcome were not always observed using BMI (a proxy for body fatness) as the outcome.^36, 37^ While the 100 c.p.m. accelerometer cut point to define ST may have included some standing time, the objective measurement of sedentary behavior using the Actigraph can be seen as a strength. A recent study comparing several cut points against a posture based monitor concluded the 100 c.p.m. cut point is most appropriate when measuring sedentary behavior in children.^38^ In addition, the inclusion of fragmentation of sedentary behavior as an outcome is novel and a benefit of this method is that it corrects for differences in sedentary time between participants. However, the method used (that is, bouts per sedentary hour) also has its limitations. Using the number of bouts does not account for the duration in which these bouts have been accumulated which may impact the results. It is recommended that future studies examine the way in which these bouts are accumulated and whether for example accumulating 25 min of sedentary time in five, 5 min bouts has a different effect on health compared to accumulating sedentary time in five 1 min bouts and one 20 min bout. The authors acknowledge that using bio-electrical impedance to measure body fat has its own limitations. Measures may have been affected by exercise or consuming food/drinks beforehand. As previously mentioned, the *post hoc* linear regression analyses were cross-sectional which is a limitation. Due to sample size it was not possible to use bivariate spline modeling to look at average change rates over specific time points (that is, the *post hoc* analysis) and therefore had to use linear regression analysis which decreased the number of participants included in these analyses. A weakness of the current study was the measure used to determine socio-economic status which relies on 1991 Census data and was measured at birth. The child’s socio-economic environment might therefore have changed by the time accelerometer data were collected. Generalizability of study findings is unclear, but we note that the cohort is both reasonably contemporary (born in 1999–2000), and socio-economically representative of North East England,^19^ and both of these characteristics should enhance generalizability.

## Conclusions

In this study a greater increase in sedentary time and increase in sedentary fragmentation from age 7–15 years were associated with increased adiposity. However, the associations differed between time points—an increase in fragmentation of sedentary behavior was associated with a decrease in adiposity from age 9–12 years. The steepest increase in adiposity associated with sedentary behavior was noted over the 9–12 years period. This is the first study to show an association between sedentary behavior and adiposity and highlights a potentially crucial period, that of late childhood to intervene to reduce the impact of age-related changes in sedentary behavior on body fatness.

## Figures and Tables

**Table 1:** Summary of the participants at each follow-up period

		*Follow-up period*			
		*7 years*	*9 years*	*12 years*	*15 years*
Sample	*n* (% with valid data)	502 (97)	506 (98)	420 (95)	306 (94)
Sex (male)	%	50.2	48.2	46.9	46.7
Age (years)	Mean (s.d.)	7.5 (0.5)	9.3 (0.4)	12.5 (0.3)	15.2 (0.4)
Weight (kg)	Mean (s.d.)	26.4 (5.2)	33.5 (7.6)	49.6 (12.2)	62.2 (14.1)
Height (cm)	Mean (s.d.)	124.9 (5.6)	135.6 (6.4)	154.6 (7.8)	167.0 (8.3)
Townsend score (*n*)	*n* (%)				
	1 (most affluent)	97 (19.3)	96 (18.9)	85 (20.2)	59 (19.3)
	2	113 (22.5)	119 (23.5)	101 (24.1)	79 (25.9)
	3	108 (21.5)	110 (21.7)	94 (22.4)	68 (22.3)
	4	90 (17.9)	93 (18.4)	68 (16.2)	48 (15.7)
	5 (least affluent)	88 (17.5)	82 (16.2)	66 (15.7)	49 (16.1)
	Missing	6 (1.2)	6 (1.2)	6 (1.4)	3 (0.7)
BMI (kg m^−^^2^)	Mean (s.d.)	16.8 (2.3)	18.0 (2.9)	20.6 (3.9)	22.2 (4.4)
FMI (kg m^−^^2^)^a^	Mean (s.d.)	4.0 (1.8)	4.8 (2.4)	5.4 (3.3)	6.5 (4.1)
Wear time per day (min)	Mean (s.d.)	671.1 (68.9)	672.5 (75.6)	717.7 (82.6)	725.6 (82.5)
Number of valid days included	Mean (s.d.)	6.4 (0.9)	6.0 (1.2)	5.9 (1.3)	5.6 (1.3)
ST (%)	Mean (s.d.)	51.5 (7.7)	55.4 (7.0)	64.8 (8.2)	73.4 (6.6)
ST (min)	Mean (s.d.)	346.5 (66.4)	372.8 (63.3)	466.5 (86.6)	535.1 (85.6)
SF (bouts/sedentary hour)	Mean (s.d.)	16.7 (1.7)	16.7 (1.6)	15.3 (2.3)	13.0 (2.4)
Accumulated time spent in sedentary bouts >1 min	Mean (s.d.)	263.3 (65.9)	294.0 (64.1)	394.8 (88.0)	480.6 (88.9)
MVPA (%)	Mean (s.d.)	6.0 (2.6)	5.5 (2.3)	4.4 (2.4)	4.3 (2.4)

Abbreviations: BMI, body mass index; FMI, fat mass index; MVPA, percentage of daily time spent in moderate-to-vigorous physical activity; SF, fragmentation of sedentary time; ST, percentage of daily time spent sedentary.

a Smaller sample due to missing data (7 years *n* 500, 9 years *n* 424; 12 years *n* 378; 15 years *n* 298).

**Table 2:** Mean differences between the change in sedentary behavior (predictor) and the change in adiposity (outcome), adjusted for sex, MVPA and seasonality (*n* 659)

*Bivariate model*	*Co-efficient*	*95% CI*		P*-value*
*Model 1 (ST and BMI)*				
ST 7 and BMI 7 years	−0.05	−0.07	−0.03	<0.001
ST 7 years and BMI Δ	−0.02	−0.03	−0.01	<0.001
ST Δ and BMI Δ	0.08	0.06	0.10	<0.001
				
*Model 2 (ST and FMI)*				
ST 7 and FMI 7 years	−0.05	−0.07	−0.04	<0.001
ST 7 years and FMI Δ	−0.02	−0.03	−0.01	<0.001
ST Δ and FMI Δ	0.15	0.11	0.19	<0.001

*Model 3 (SF and BMI)*				
SF 7 and BMI 7 years	−0.002	−0.11	0.11	0.968
SF 7 years and BMI Δ	0.04	−0.06	0.15	0.409
SF Δ and BMI Δ	0.07	0.06	0.09	<0.001
				
*Model 4 (SF and FMI)*				
SF 7 and FMI 7 years	−0.01	−0.12	0.11	0.927
SF 7 years and FMI Δ	0.05	−0.06	0.15	0.396
SF Δ and FMI Δ	0.14	0.10	0.18	<0.001

Abbreviations: BMI, body mass index; CI, confidence interval; FMI, fat mass index; MVPA, percentage of daily time spent in moderate-to-vigorous physical activity; SF, fragmentation of sedentary time; ST, percentage of daily time spent sedentary.

Δ: change from 7 to 15 years of age; Δ change between time points.

**Table 3:** Linear regression model of change in ST or SF (predictors) and change in BMI (outcome) between time points, adjusted for change in MVPA and sex

*BMI*	*Co-efficient*	*95% CI*		P*-value*
ST Δ 7 and 9 years (*n* 400)	−0.02	−0.04	0.00	0.053
ST Δ 9 and 12 years (*n* 348)	0.02	−0.02	0.05	0.316
ST Δ 12 and 15 years (*n* 260)	0.01	−0.03	0.06	0.594
SF Δ 7 and 9 years (*n* 400)	0.06	0.00	0.13	0.049
SF Δ 9 and 12 years (*n* 348)	−0.09	−0.18	0.00	0.046
SF Δ 12 and 15 years (*n* 260)	0.05	−0.05	0.15	0.322

Abbreviations: BMI, body mass index; CI, confidence interval; MVPA, percentage of daily time spent in moderate-to-vigorous physical activity; SF, fragmentation of sedentary time; ST, percentage of daily time spent sedentary.

Δ change between time points.

**Table 4:** Linear regression model of change in ST or SF (predictor) and change in FMI (outcome) between time points, adjusted for change in MVPA and sex.

*FMI*	*Co-efficient*	*95% CI*		P*-value*
ST Δ 7 and 9 years (*n* 374)	0.00	−0.02	0.02	0.703
ST Δ 9 and 12 years (*n* 275)	0.04	0.00	0.07	0.062
ST Δ 12 and 15 years (*n* 232)	0.02	−0.04	0.07	0.529
SF Δ 7 and 9 years (*n* 374)	0.09	−0.04	0.23	0.174
SF Δ 9 and 12 years (*n* 275)	−0.11	−0.22	0.00	0.049
SF Δ 12 and 15 years (*n* 232)	0.07	−0.05	0.19	0.246

Abbreviations: CI, confidence interval; FMI, fat mass index; MVPA, percentage of daily time spent in moderate-to-vigorous physical activity; SF, fragmentation of sedentary time; ST, percentage of daily time spent sedentary.

Δ change between time points.
