# Supplementary material for: Longitudinal study of the associations between change in sedentary behavior and change in adiposity during childhood and adolescence: Gateshead Millennium Study
Source: Int J Obes (Lond). 2017 May 9;41(7):1042–7. doi: 10.1038/ijo.2017.69 (PMC5500163; doi:10.1038/ijo.2017.69)
Supplement: Supplementary Table 1 [file ijo201769x1.docx]

Online Supplement 1: Linear spline model estimates for the mean trajectories of change in sedentary behavior (sedentary time and fragmentation) and adiposity (BMI and FMI) between 7y and 15y.

| **Exposure variable** | **Mean predicted (95% CI) intercept and slopes in boys** | | | **p-value** | **Mean predicted  (95% CI ) intercept and slopes in girls** | | | **p-value** |
| --- | --- | --- | --- | --- | --- | --- | --- | --- |
| **Sedentary time model^1^** |  |  | |  |  |  |  |  |
| ST (%) at 7y | 58.35 | (56.04, | 60.67) | <0.001 | 60.87 | (58.68, | 63.05) | <0.001 |
| ST change 7y – 9y | 0.81 | (-0.62, | 2.24) | 0.268 | 0.56 | (-0.73, | 1.85) | 0.379 |
| ST change 9y – 12y | 3.64 | (2.85, | 4.43) | <0.001 | 3.45 | (2.78, | 4.12) | <0.001 |
| ST change 12y – 15y | 2.71 | (1.84, | 3.58) | <0.001 | 2.81 | (2.04, | 3.58) | <0.001 |
| **Sedentary fragmentation model^1^** | |  |  |  |  |  |  |  |
| SF (bph) at 7y | 15.49 | (14.77 | 16.21) | <0.001 | 15.12 | (14.43, | 15.81) | <0.001 |
| SF change 7y – 9y | 0.68 | (0.19 | 1.16) | 0.006 | 0.38 | (-0.06, | 0.81) | 0.089 |
| SF change 9y – 12y | -0.53 | (-0.81 | -0.24) | <0.001 | -0.43 | (-0.67, | -0.19) | <0.001 |
| SF change 12y – 15y | -0.76 | (-1.11 | -0.42) | <0.001 | -0.98 | (-1.28, | -0.67) | <0.001 |
| **BMI model^2^** |  |  |  |  |  |  |  |  |
| BMI (kg/m^2^) at 7y | 16.73 | (16.22 | 17.23) | <0.001 | 16.72 | (16.26, | 17.18) | <0.001 |
| BMI change 7y – 9y | 0.77 | (0.47 | 1.06) | <0.001 | 0.96 | (0.70, | 1.21) | <0.001 |
| BMI change 9y – 12y | 0.92 | (0.73 | 1.10) | <0.001 | 0.90 | (0.75, | 1.04) | <0.001 |
| BMI change 12y – 15y | 0.41 | (0.22 | 0.60) | <0.001 | 0.75 | (0.60, | 0.89) | <0.001 |
| **FMI model^2^** |  |  |  |  |  |  |  |  |
| FMI (kg/m^2^) at 7y | 4.72 | (4.24 | 5.19) | <0.001 | 4.19 | (3.76, | 4.62) | <0.001 |
| FMI change 7y – 9y | 0.56 | (0.23 | 0.88) | 0.001 | 0.85 | (0.56, | 1.13) | <0.001 |
| FMI change 9y – 12y | 0.09 | (-0.13 | 0.30) | 0.435 | 0.00 | (-0.18, | 0.17) | 0.960 |
| FMI change 12y – 15y | -0.19 | (-0.40 | 0.02) | 0.071 | 0.84 | (-0.07, | 0.25) | <0.001 |

95%CI: 95% Confidence Interval; ST: Sedentary time; SF: Sedentary fragmentation; bph; bouts per sedentary hour; BMI: Body Mass Index; FMI: Fat Mass Index

^1^ All estimates adjusted for MVPA, sex, season and an interaction between sex and MVPA.

^2^ All estimates adjusted for MVPA and sex.
