# Supplementary material for: Longitudinal study of the associations between change in sedentary behavior and change in adiposity during childhood and adolescence: Gateshead Millennium Study
Source: Int J Obes (Lond). 2017 May 9;41(7):1042–7. doi: 10.1038/ijo.2017.69 (PMC5500163; doi:10.1038/ijo.2017.69)
Supplement: Supplementary Table 4 [file ijo201769x4.docx]

Online Supplement 4: Quantile regression models of sedentary fragmentation (SF) and the change in body mass index (BMI) percentiles from ages 7 to 15 years.

|  | ***BMI*** | | | | |
| --- | --- | --- | --- | --- | --- |
|  | ***10th Percentile*** | ***25th Percentile*** | ***50th Percentile*** | ***75th Percentile*** | ***90th Percentile*** |
| *Model 1* | | | | | |
| Intercept | 14.5  (14.29, 14.71) | 15.2  (15.00, 15.40) | 16.2  (16.00, 16.40) | 17.7  (17.32, 18.08) | 19.86  (19.25, 20.47) |
| Time | 0.23  (0.10, 0.36) | 0.48  (0.37, 0.59) | 0.72  (0.56, 0.87) | 1.05  (0.87, 1.23) | 1.27  (0.93, 1.60) |
| Time^2^ | 0.03  (0.01, 0.04) | 0.00  (-0.01, 0.02) | 0.00  (-0.02, 0.02) | -0.03  (-0.05, 0.00) | -0.03  (-0.08, 0.02) |
| *Model 2* | | | | | |
| Intercept | 15.07  (13.07, 17.08) | 16.74  (15.31, 18.16) | 19.18  (17.63, 20.72) | 21.45  (18.32, 24.59) | 23.40  (19.56, 27.53) |
| Time | 0.23  (0.09, 0.37) | 0.46  (0.40, 0.52) | 0.61  (0.54, 0.68) | 0.78  (0.67, 0.89) | 1.00  (0.80, 1.20) |
| Time^2^ | 0.02  (0.00, 0.05) | NA | NA | NA | NA |
| SF | -0.03  (-0.15, 0.09) | -0.09  (-0.17, -0.01) | -0.17  (-0.26, -0.09) ^#^ | -0.22  (-0.40, -0.04) ^#^ | -0.20  (-0.44, 0.04)^#^ |
| *Model ** | | | | | |
| Intercept | 14.89  (12.83, 16.96) | 16.40  (14.77, 18.02) | 18.13  (16.26, 20.00) | 21.00  (17.79, 24.21) | 24.90  (21.45, 28.35) |
| Time | 0.26  (0.11, 0.40) | 0.46  (0.40, 0.52) | 0.59  (0.52, 0.65) | 0.73  (0.61, 0.84) | 0.91  (0.74, 1.09) |
| Time^2^ | 0.02  (0.00, 0.04) | NA | NA | NA | NA |
| SF | -0.05  (-0.17, 0.06) | -0.06  (-0.14, 0.02) | -0.11  (-0.20, -0.02) ^#^ | -0.11  (-0.27, 0.05) | -0.14  (-0.34, 0.07) |

*model adjusted for MVPA and sex; ^#^p<0.05.

SF, sedentary fragmentation. Model 1 describes change in BMI over time per percentile. Model 2: as model 1 with inclusion of SF as independent variable. Model 3: as Model 2 with inclusion of MVPA and sex as covariates. Data presented are coefficients (95% confidence intervals). Time is coded 0, 2, 5 and 8 and time^2^ is coded 0, 4, 25 and 64 for age 7, 9, 12 and 15 years, respectively. The sedentary fragmentation coefficients are the changes in BMI, at each percentile, for every additional bout spent in sedentary behavior per hour of sedentary time.
